# Circulating mediators of bone remodeling in psoriatic arthritis: implications for disordered osteoclastogenesis and bone erosion

**DOI:** 10.1186/ar3123

**Published:** 2010-08-26

**Authors:** Nicola Dalbeth, Bregina Pool, Timothy Smith, Karen E Callon, Maria Lobo, William J Taylor, Peter B Jones, Jillian Cornish, Fiona M McQueen

**Affiliations:** 1 Department of Medicine, University of Auckland, 85 Park Rd, Auckland 1010, New Zealand; 2 Department of Rheumatology, Auckland District Health Board, Greenlane West, Auckland 1051, New Zealand; 3 Department of Medicine, University of Otago, Wellington, Mein St, Wellington 6021, New Zealand; 4 Waikato Clinical School, University of Auckland, Pembroke Street, Hamilton 3240, New Zealand; 5 Department of Molecular Medicine and Pathology, University of Auckland, 85 Park Rd, Auckland 1010, New Zealand

## Abstract

**Introduction:**

Diverse bone pathologies are observed in patients with psoriatic arthritis (PsA). Uncoupling of bone remodeling with disordered osteoclastogenesis has been implicated in the pathogenesis of PsA. The aim of this study was to examine the role of soluble mediators of bone remodeling within the circulation of patients with PsA.

**Methods:**

Patients with PsA (*n *= 38), with psoriasis (*n *= 10), and healthy controls (*n *= 12) were studied. Serum was obtained for testing of Dikkopf-1 (Dkk-1), macrophage-colony stimulating factor (M-CSF), osteoprotegerin (OPG), and receptor activator of nuclear factor-κB ligand (RANKL) with ELISA. Patients with PsA also had bone densitometry, plain radiographs of the hands and feet, and assessment of peripheral blood osteoclast precursors. Radiographs were scored for erosion, joint-space narrowing, osteolysis, and new bone formation.

**Results:**

Compared with those with psoriasis and healthy controls, patients with PsA had higher circulating concentrations of Dkk-1 and M-CSF. In patients with PsA, M-CSF and RANKL, but not Dkk-1, concentrations positively correlated with radiographic erosion, joint-space narrowing, and osteolysis scores. Mediators of bone remodeling did not correlate with the number of joints with new bone formation or with total hip-bone mineral density. Peripheral blood CD14^+^/CD11b^+ ^cells, and the number of osteoclast-like cells and resorptive pits after culture with RANKL and M-CSF also correlated with radiographic damage scores. Circulating M-CSF concentrations correlated with the percentage of peripheral blood CD14^+^/CD11b^+ ^cells.

**Conclusions:**

Systemic expression of soluble factors that promote osteoclastogenesis is disordered in patients with PsA and may contribute to periarticular bone loss in this disease.

## Introduction

Psoriatic arthritis (PsA) is an inflammatory arthritis with a number of characteristic clinical features [1]. PsA is typically associated with psoriasis and psoriatic nail disease and has both peripheral articular manifestations (including synovitis, dactylitis, and enthesitis) and axial skeletal involvement. A range of bone pathologies is observed in patients with PsA [2]. Bone loss can occur, either locally in the form of bone erosion and osteolysis affecting the peripheral joints, or systemically with loss of skeletal bone mineral density (BMD) [3]. Aberrant bone formation may also occur, including peripheral juxtaarticular new bone formation, ankylosis, and syndesmophyte formation. These different bone pathologies may be observed in the same patient [4].

Bone is a metabolically active tissue that is capable of constantly remodeling in a highly coordinated manner (reviewed in [5]). Two main cell types are involved in bone remodeling: osteoclasts that resorb mineralized bone and osteoblasts that are responsible for new bone formation. Osteoclasts can be matured *in vitro *by culture of monocyte/macrophage precursors in the presence of macrophage-colony stimulating factor (M-CSF) and receptor activator of nuclear factor-κB ligand (RANKL) [6,7]. Osteoprotegerin (OPG) is a further mediator of bone remodeling, acting as a decoy receptor that prevents RANKL binding to its receptor RANK, thus inhibiting osteoclastogenesis [7]. When cultured on bone, osteoclasts derived *in vitro *excavate resorptive pits that are similar to the structures formed when osteoclasts degrade bone *in vivo*. These cells also express proteins that typify the osteoclast lineage, including tartrate-resistant acid phosphatase (TRAP). Additional soluble factors also influence bone remodeling; recent work has demonstrated that Dickkopf (Dkk-1), an inhibitor of Wnt signaling, inhibits osteoblast differentiation and function and promotes osteoclastogenesis through suppression of OPG [8-11].

RANKL-mediated osteoclastogenesis has been implicated in the pathogenesis of bone resorption in PsA [12-15]. In patients with PsA, osteoclasts are present at sites of bone erosion, and osteoclasts cultured *in vitro *from peripheral blood precursors exhibit increased resorptive activity compared with those from healthy controls [12]. Erosive disease is also associated with high numbers of circulating cells expressing CD14 and CD11b [12]. Intense RANKL expression has been demonstrated within the lining layer in the PsA joint, with more restricted sublining layer OPG expression, implicating imbalance in the RANKL/OPG axis that, in turn, may promote osteoclastogenesis [12]. However, the factors regulating the various manifestations of bone disease in PsA remain uncertain.

The aim of this study was to examine the role of soluble mediators of bone remodeling in the circulation of patients with PsA. Here, we focused on four soluble mediators that have been definitively implicated in bone remodeling in models of inflammatory arthritis; Dkk-1, M-CSF, RANKL, and OPG [11,16-19]. In particular, we wished to determine the relations between these mediators and patterns of bone pathology in PsA.

## Materials and methods

### Patients and clinical assessments

This study was approved by the New Zealand Multiregion Ethics Committee, and all patients provided written informed consent. Thirty-eight patients with PsA were recruited from rheumatology clinics in the Auckland, Rotorua, and Wellington regions of New Zealand. All patients with PsA met the Classification of Psoriatic Arthritis (CASPAR) criteria for PsA [1]. In addition, two control groups were studied; patients with psoriasis (confirmed by a dermatologist) but no arthritis (*n *= 10), and healthy volunteers with no psoriasis or arthritis (*n *= 12). Psoriasis control and healthy control participants had no previous diagnosis of arthritis and no evidence of synovitis, enthesitis, joint deformity, or spinal limitation on physical examination at the time of recruitment.

Clinical assessments, radiographs, and blood samples were completed at a single study visit. All participants, including healthy control and psoriasis control participants, had collection of demographic data, recording of relevant medical history and medications, and serum measured for soluble mediators of bone remodeling and C-reactive protein. Severity of psoriasis was assessed in patients with psoriasis and PsA by using the Psoriasis Area and Severity Index (PASI) [20], and psoriatic nail disease was assessed by using the Psoriasis Nail Severity Score (PNSS) [21]. Patients with PsA also had further investigations, including assessment of arthritis disease activity by using the disease activity score (DAS)28-CRP, bone densitometry, plain radiographs of the hands, feet, and sacroiliac joints, and assessment of peripheral blood osteoclast precursors.

### Radiographic assessments

Plain radiographs of the hands, feet, and sacroiliac joints were obtained at the study visit. Plain radiographs of the hands and feet were scored for erosions and joint-space narrowing according to the Sharp van der Heijde score modified for use in PsA [22], by a rheumatologist (ND) with experience in this scoring system. Patients with at least one erosion on hand and foot radiographs were considered erosive for the purposes of the analysis. The number of joints with new bone formation was recorded by using the CASPAR definition: radiographic evidence of juxtaarticular new bone formation appearing as ill-defined ossification near joint margins (but excluding osteophyte formation) on plain radiographs of the hand or foot [1]. The number of joints in the hands and feet with pencil-in-cup deformities was also recorded to allow a quantitative assessment of the presence and extent of osteolysis. Sacroiliitis was scored as present or absent by a radiologist, according to the New York criteria for sacroiliitis in ankylosing spondylitis. Proximal femur BMD was measured by using a Prodigy dual-energy x-ray absorptiometer (DEXA) (GE-Lunar, Madison, WI). All radiographic scoring and measurement were completed by readers who were blinded to the clinical and laboratory findings.

### Testing of soluble mediators of bone remodelling

Blood was obtained at the study visit, and serum was separated within 3 hours of collection. Serum was separated into at least four aliquots to avoid repeated freeze-thaw cycles and was stored at -20°C until testing. Serum was analyzed for soluble mediators of bone remodeling with enzyme-linked immunosorbent assay (ELISA) by using the following kits; Dkk-1 (R&D duoset), M-CSF (R&D quantikine), and RANKL (Biomedica), OPG (R&D duoset), according to the manufacturers' instructions. To confirm consistency between assays, three control sera were used as internal controls for each ELISA plate. Initial optimization assays of the Dkk-1 assay confirmed significantly elevated concentrations of Dkk-1 in a patient with osteolytic metastatic bone disease (16,527 pg/ml).

### Testing of peripheral blood osteoclast precursors

Peripheral blood mononuclear cells (PBMCs) were isolated with Lymphoprep (Nycomed Pharma, Oslo, Norway) gradient centrifugation. The cells were analyzed for the presence of osteoclast precursors with flow cytometry and culture in RANKL and M-CSF, as previously described [23].

Osteoclast precursors arise from the CD14^+^/CD11b^+ ^monocyte population [24]. The percentage of CD14^+^/CD11b^+ ^cells was studied with flow-cytometry analysis of peripheral blood [12,25]. PBMCs (10^6 ^cells/assay) were washed in phosphate-buffered saline (PBS) in the presence of 0.16% bovine serum albumin and 0.1% sodium azide and incubated for 30 minutes with saturating amounts of the anti-CD14-fluorescein isothiocyanate (FITC) (Dako, Carpinteria, CA) and anti-CD11b-phycoerythrin (PE) (Dako), or appropriate fluorescein-conjugated isotype control antibodies. All samples were analyzed on a FACScan by using Cell Quest Software (both from Becton Dickinson, Mountain View, CA). The percentage of CD14^+^/CD11b^+ ^cells in each PBMC sample was recorded.

PBMCs (10^6 ^cells/ml) were placed in 24-well plates containing 1 ml αMEM with 10% fetal bovine serum (FBS), 100 units/ml penicillin, and 100 μg/ml streptomycin. Cells were incubated at 37°C in 5% CO_2 _for 14 days with and without human recombinant RANKL (30 ng/ml; Peprotech Ltd, Rehovot, Israel) and M-CSF (25 ng/ml, R&D Inc., Minneapolis, MN). Medium was replenished every 3 to 4 days. After 14 days in culture, slides were stained for TRAP (Sigma, Poole, UK). Slides were viewed with light microscopy, and TRAP-positive cells with three or more nuclei were counted as osteoclasts by a single observer who was blinded to the clinical and radiographic characteristics of the patients. Cells were plated and counted in triplicate, and the mean value of the triplicates was recorded.

The ability of these cells to resorb bone was confirmed in parallel assays, by culturing of PBMCs in 1 ml 10% FBS-αMEM with RANKL and M-CSF for 14 days on ivory slices in duplicate (supplied by the Auckland Conservancy Office, Department of Conservation, Auckland, New Zealand). The cultured ivory slices were scrubbed, stained with toluidine blue, and analyzed for resorption pits with reflected-light microscopy. The number of pits on the entire slice was counted manually by a single observer who was blinded to the clinical and radiographic characteristics of the patients. The mean value of the duplicates was recorded.

### Statistical analysis

All data were analyzed by using GraphPad Prism (GraphPad Software, San Diego, CA). Descriptive data are presented as *n *(percentage) or median (range). Differences between groups was analyzed with χ^2 ^tests and Mann-Whitney tests in the case of two groups, and one-way analysis of variance (ANOVA) (Kruskal-Wallis test) with Dunn's multiple comparison test in the case of more than two groups. Spearman's correlations were used to explore the relation between the clinical/radiographic features and laboratory results. A *P *value of < 0.05 was considered significant.

## Results

### Clinical characteristics

Of the 38 patients with PsA, 29 had at least one erosion on plain radiography (erosive), and nine had no erosions (nonerosive). Clinical characteristics of the patients with PsA, of those with psoriasis, and of healthy controls are shown in Table 1. All groups were matched by age and ethnicity. Some differences were observed between groups. More women were in the healthy control group. Psoriasis control participants had higher PASI scores and less use of methotrexate and nonsteroidal antiinflammatory drugs (NSAIDs), compared with the PsA group. Both psoriasis and healthy controls had lower C-reactive protein concentrations than did patients with PsA. Patients with erosive and nonerosive PsA were similar, except for higher DAS28-CRP and radiographic-damage scores in the erosive group. No patients were receiving TNF inhibitors or other biologic therapy.

**Table 1:** Clinical characteristics of study participants

	All PsA *n *= 38	Erosive PsA *n *= 29	Nonerosive PsA *n *= 9	Psoriasis alone *n *= 10	Healthy control *n *= 12
Female sex, *n *(%)	16 (42%)	11 (38%)	5 (56%)	4 (40%)	11 (92%)^b^
Age, years, median (range)	50 (26-68)	50 (26-63)	47 (33-68)	44 (19-72)	48 (21-53)
Caucasian ethnicity, *n *(%)	32 (84%)	25 (86%)	7 (78%)	7 (70%)	10 (83%)
Weight, kg, median (range)	78 (32-116)	80 (65-114)	74 (32-116)	82 (67.9-103)	81 (46-109)
Psoriasis disease duration, years, median (range)	20 (0.5-50)	20 (3-50)	20 (0.5-40)	18 (0.4-53)	NA
Arthritis disease duration, years, median (range)	10 (0.5-45)	11 (5-33)	5 (0.5-45)	NA	NA
PASI, median (range)	1.6 (0-12)	1.5 (0-12)	1.6 (1-3.4)	13 (6.3-23.2)^b^	NA
Nail score, median (range)	7 (0-47)	9 (0-47)	6 (0-25)	6 (0-25)	NA
Creatinine, μmol/L, median (range)	77 (23-249)	78 (23-249)	77 (42-89)	76 (39-100)	NA
C-reactive protein, mg/L, median (range)	6.5 (1-59)	7.2 (1-59)	5.8 (1-26)	2.6 (1-7.2)^b^	2.1 (1-15)^b^
DAS28-CRP, median (range)	3.9 (1.4-6.6)	4.2 (2.3-6.6)	2.7 (1.4-6.0)^a^	NA	NA
Methotrexate use, *n *(%)	22 (58%)	15 (52%)	7 (78%)	1 (10%)^b^	0 (0)^b^
Prednisone use, *n *(%)	7 (18%)	4 (14%)	3 (33%)	0 (0)	0 (0)
Nonsteroidal antiinflammatory drug use, *n *(%)	19 (50%)	16 (55%)	3 (33%)	0 (0)^b^	0 (0)^b^
Biologics use, *n *(%)	0 (0)	0 (0)	0 (0)	0 (0)	0 (0)
XR erosion score, median (range)	29 (0-183)	43 (1-183)	0 (0-0)^a^	NA	NA
XR narrowing score, median (range)	24.5 (0-160)	36 (2-160)	0 (0-10)^a^	NA	NA
Combined XR score, median (range)	59 (0-343)	78 (3-343)	0 (0-10)^a^	NA	NA
Number of joints with pencil-in-cup deformities on XR, median (range)	0 (0-17)	0 (0-17)	0 (0-0)	NA	NA
Number of joints with new bone formation on XR, median (range)	1 (0-9)	1 (0-9)	0 (0-7)	NA	NA
Radiographic sacroiliitis, *n *(%)	15 (39%)	12 (41%)	3 (33%)	NA	NA
Total hip BMD *T *score, median (range)	-0.1 (-3.5-2.0)	-0.1 (-2.1-2.0)	-0.2 (-3.5-0.9)	NA	NA

^a^*P *< 0.05, compared with erosive PsA; ^b^*P *< 0.05, compared with all PsA. BMD, bone mineral density DAS, disease activity score; PASI, Psoriasis Area and Severity Index; XR, x-ray. NA, not assessed.

### Soluble mediators of bone remodeling in the circulation of patients with PsA

**Figure 1:**
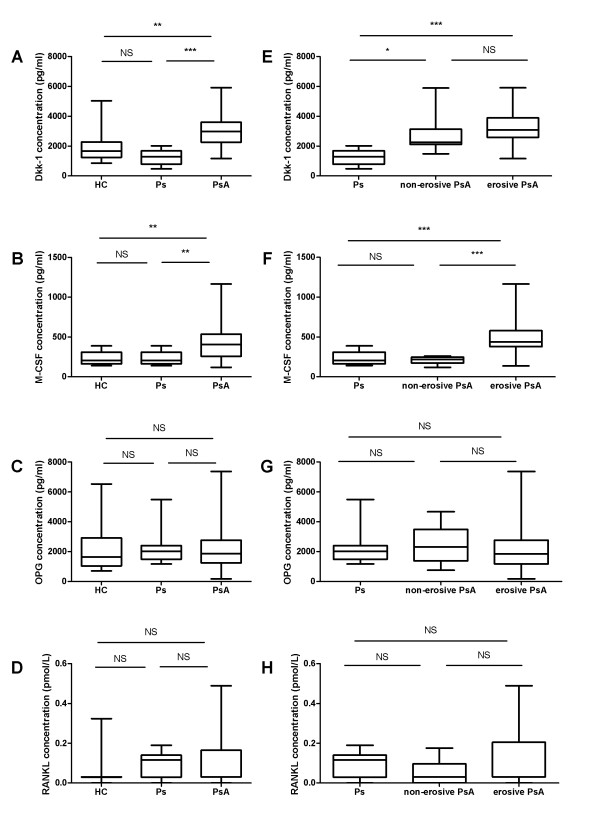
**Soluble mediators of bone remodeling in the circulation of patients with PsA**. Box-and-whisker plots showing concentrations of **(a) **Dkk-1, **(b) **M-CSF, **(c) **OPG, and **(d) **RANKL in healthy controls (HCs), patients with psoriasis (Ps) and patients with PsA. Box-and-whisker plots showing concentrations of **(e) **Dkk-1, **(f) **M-CSF, **(g) **OPG, and **(h) **RANKL in patients with psoriasis (Ps) and patients with nonerosive PsA, and patients with erosive PsA. Median values for RANKL for the healthy control, all PsA, and erosive PsA groups were 0.031 pmol/L. *P < 0.05; **P < 0.01; ***P < 0.001; one-way ANOVA with Dunn's multiple comparison test.

Compared with both healthy controls and psoriasis controls, patients with PsA had higher circulating concentrations of Dkk-1 and M-CSF (Figure 1a and 1b). No significant difference was found between the control groups and the PsA group in OPG or RANKL concentrations (Figure 1c and 1d).

Patients with both erosive and nonerosive PsA had higher circulating concentrations of Dkk-1, compared with psoriasis controls (Figure 1e). In contrast, only those with erosive PsA had higher M-CSF concentrations (Figure 1f). No overall difference was noted in RANKL or OPG concentrations between the groups of patients with erosive and nonerosive PsA (Figure 1g and 1h).

### Relation between soluble mediators of bone remodeling and patterns of bone disease in patients with PsA

In patients with PsA, M-CSF and RANKL concentrations positively correlated with radiographic-damage scores, including erosion scores, joint-space narrowing scores, and the number of joints affected by osteolysis (Table 2). No relation was observed between Dkk-1 concentrations and patterns of bone disease in these patients. Circulating mediators of bone remodeling did not correlate with the number of joints affected by new bone formation or total hip BMD (including after the adjustment for BMI; data not shown). In patients with PsA, no relation was observed between the soluble mediators of bone remodeling and the following clinical factors: sex, creatinine, weight, presence of sacroiliitis, DAS-28-CRP, or prednisone, or NSAID or methotrexate use (data not shown).

**Table 2:** Stromal cell-derived mediators of bone remodeling and bone pathology scores in patients with PsA

	Median (range)	XR erosion score	XR narrowing score	Combined XR score	Number of joints with pencil-in-cup deformities on XR	Number of joints with new bone formation on XR	Total hip BMD *T *score
Dkk-1	2,977 (1,163-5,908) pg/ml	0.15	0.10	0.15	-0.12	0.22	-0.13
M-CSF	408 (120-1,166) pg/ml	0.53^b^	0.54^b^	0.52^b^	0.36^a^	0.09	-0.06
OPG	1,871 (179.5-7,367) pg/ml	-0.13	-0.03	-0.08	0.09	-0.12	-0.10
RANKL	0.031 (0-0.49) pmol/L	0.36^a^	0.35^a^	0.36^a^	0.41^a^	0.08	-0.03

^a^*P *< 0.05; ^b^*P *< 0.01. Spearman *r *values for correlations between circulating concentrations of stromal cell-derived mediators of bone remodeling and measures of bone pathology. BMD, bone mineral density; Dkk-1, Dikkopf-1; M-CSF, macrophage-colony stimulating factor; OPG, osteoprotegerin; RANKL, receptor activator of nuclear factor-κB ligand; XR, x-ray.

### Peripheral blood osteoclast precursors, patterns of bone disease, and soluble mediators of bone remodeling in patients with PsA

In patients with PsA, the percentage of CD14^+^/CD11b^+ ^cells in peripheral blood, the number of TRAP^+ ^multinucleated cells (MNCs), and pits after culture with RANKL and M-CSF correlated with radiographic damage scores (Table 3). The number of joints affected by osteolysis also correlated with the number of TRAP^+ ^MNCs and pits after culture (Table 3). The number of pits strongly correlated with the number of TRAP^+ ^MNCs after culture with RANKL and M-CSF (*r *= 0.53; *P *= 0.001), but not with the total percentage of CD14+/CD11b+ cells (*r *= 0.29; *P *= 0.09). After adjusting for the number of CD14^+^/CD11b^+ ^cells, the correlation with TRAP^+ ^MNCs was no longer significant, but a persistent association was found with the number of pits in culture and radiographic damage and osteolysis (Table 3). Large numbers of spontaneously arising bone-resorbing osteoclast-like cells were not observed in patients with PsA or in control participants in the absence of RANKL and M-CSF.

**Table 3:** Cellular markers of bone remodeling and bone pathology scores in patients with PsA

	Median (range)	XR erosion score	XR narrowing score	Combined XR score	Number of joints with pencil-in-cup deformities on XR	Number of joints with new bone formation on XR	Total hip BMD *T *score
Percentage CD11b^+^/CD14^+ ^cells (% all PBMCs)	3.3 (0.04-9.0)	0.35^a^	0.36^a^	0.38^a^	0.11	0.03	-0.20
TRAP^+ ^MNCs (per 10^6 ^PBMCs)^c^	29 (0-913)	0.31	0.41^a^	0.37^a^	0.34^a^	0.00	-0.18
TRAP^+ ^MNCs adjusted for number of CD11b^+^/CD14^+ ^cells^c^	9 (0-454)	0.18	0.21	0.17	0.28	0.00	-0.10
Number of pits (per 10^6 ^PBMCs)	0 (0-500)	0.43^a^	0.48^b^	0.46^b^	0.41^a^	0.00	-0.33
Number of pits adjusted for number of CD11b^+^/CD14^+ ^cells^c^	0 (0-371)	0.45^b^	0.49^b^	0.47^b^	0.45^b^	0.01	-0.30

^a^*P *< 0.05; ^b^*P *< 0.01; ^c^after culture with RANKL and M-CSF. Spearman *r *values for correlations between cellular markers and measures of bone pathology. BMD, bone mineral density; M-CSF, macrophage-colony stimulating factor; MNCs, multinucleated cells; PBMCs, peripheral blood mononuclear cells; RANKL, receptor activator of nuclear factor-κB ligand; TRAP, tartrate-resistant acid phosphatise; XR, x-ray.

Circulating M-CSF concentrations also correlated with the percentage of peripheral blood CD14^+^/CD11b^+ ^cells in patients with PsA (*r *= 0.40; *P *< 0.05), with a trend to correlation with the number of TRAP^+ ^MNCs (*r *= 0.33; *P *= 0.05). No correlation was noted between the numbers of pits after culture with M-CSF and RANKL and circulating M-CSF concentrations (*r *= 0.21; *P *> 0.05). Similarly, no correlation was observed between the other soluble mediators of bone remodeling and measures of osteoclast precursors (data not shown).

## Discussion

This study has allowed analysis of the relation between circulating factors of bone remodeling and patterns of bone pathology in PsA. The results provide further evidence for uncoupling of bone remodeling in patients with PsA. The soluble factors analyzed in this study are key regulators of bone turnover, and our data suggest that systemic expression of factors promoting osteoclastogenesis and bone loss (Dkk-1, M-CSF, RANKL) is disordered in patients with PsA.

Bone pathology in PsA may occur at a number of different sites, affecting both articular sites (erosion, osteolysis, and new bone formation) and the skeleton in a generalized manner (loss of BMD). In this study, patients with PsA were carefully characterized to identify patterns of peripheral joint bone loss and bone formation, and the relation between these patterns of bone pathology and soluble mediators of bone remodeling was analyzed. Our data indicate that the extent of bone loss at the peripheral joint is associated with elevated circulating M-CSF and RANKL concentrations, but that these factors are not associated with the extent of systemic bone loss in PsA. Furthermore, we have not identified circulating factors that are associated with peripheral new bone formation in this disease. Spinal radiographs were not obtained as part of this study, and it is possible that alterations in circulating markers of bone-remodeling factors may be associated with other forms of new bone formation in PsA, such as syndesmophyte formation in the axial skeleton. This study assessed sacroiliitis with plain radiography by using a widely recognized scoring method of established bone change. Although no relation between circulating bone-remodeling markers and sacroiliitis was observed, it is possible that that inflammation of the sacroiliac joints was underestimated by using this method, compared with a more-sensitive method such as magnetic resonance imaging [26,27].

A key finding of this study is the elevated serum Dkk-1 concentrations with patients with PsA, compared with those in patients with psoriasis and healthy control participants. Dkk-1 has been strongly implicated in joint remodeling in inflammatory arthritis; blockade of this factor inhibits osteoclastogenesis and bone erosion in *in vivo *models of RA, even in the presence of persistent joint inflammation [11]. Furthermore, blockade of Dkk-1 promotes development of osteophytes in these models, indicating a role in regulation of new bone formation [11]. Elevated serum Dkk-1 concentrations have previously been reported in patients with active RA [11]. Interestingly, we have not identified a specific relation between serum Dkk-1 concentrations and patterns of bone pathology in PsA, in the form of either new bone formation or bone loss. PsA is a heterogeneous disease, and it is possible that this study was not powered to identify differences in Dkk-1 concentrations between disease subsets, which may overlap. However, these findings are consistent with a previous study of serum Dkk-1 concentrations in inflammatory arthritis, which did not show an association with radiographic damage in rheumatoid arthritis (RA) (by using the Sharp score) or ankylosing spondylitis (by using the modified Stoke Ankylosing Spondylitis Spine Score (mSASSS)) [28]. The elevated serum Dkk-1 concentration in our study differs from that in the recent study of patients with inflammatory arthritis that included a small PsA group [28]. A further consideration is that serum Dkk-1 concentrations may not reflect the biologic activity of this mediator, particularly in the context of inflammatory disease [28]. Together, these observations indicate uncertainty about the role and significance of circulating concentrations of Dkk-1 in the development of bone disease in inflammatory arthritis.

M-CSF promotes macrophage survival and proliferation and is a key regulator of osteoclastogenesis [29]. This cytokine is required for culture of osteoclasts *in vitro *and has been strongly implicated in the pathogenesis of TNF-induced osteolysis in animal models [16,30]. We showed that circulating concentrations of M-CSF are elevated in the patients with erosive PsA and strongly correlate with severity of peripheral erosive disease. Circulating M-CSF concentrations also correlated with the percentage of peripheral blood CD14^+^/CD11b^+ ^cells in patients with PsA, but not with the number of TRAP^+ ^MNCs or the number of resorptive pits after 2-week culture with M-CSF and RANKL. These results suggest that circulating M-CSF might promote survival or release of circulating osteoclast precursor cells, rather than differentiation or activity of osteoclasts. However, it should be noted that both M-CSF and RANKL were added to the osteoclast-differentiation cultures; thus, a biologic effect of M-CSF on osteoclast development or activity *in vivo *cannot be excluded entirely.

The factors tested in this study are likely to have specific effects within the local joint environment, which may not be entirely reflected by circulating concentrations. For example, it has been reported that OPG expression is limited to endothelial cells below the synovial membrane within the psoriatic joint [12]; in contrast, we did not identify reduced circulating OPG concentrations in patients with PsA or a relation between these concentrations and patterns of bone remodeling. Our data do raise the possibility that other circulating mediators, such as M-CSF or RANKL, may have a role as biomarkers to identify patients with PsA in whom progressive or accelerated joint damage will develop. One of the key aspects of biomarker development is feasibility, and measurement of soluble markers within the circulation, rather than within synovial tissue, is clearly a major advantage in this respect [31]. Prospective studies are required to validate these factors further as biomarkers in PsA [32].

Consistent with the work of Ritchlin *et al. *[12], our study also implicates RANKL in the pathogenesis of bone erosion in PsA, noting the modest correlation between circulating RANKL concentrations and measures of peripheral-joint bone loss. Furthermore, we demonstrated that the percentage of circulating CD14^+^/CD11b^+ ^cells correlates with the extent of the erosive disease, and that osteoclasts arising from peripheral blood precursors have a greater capacity to resorb bone in those with more-severe erosive disease. It is of interest that circulating concentrations of both M-CSF and RANKL are associated with bone loss at a local level within the peripheral joints, but not with systemic BMD. Similar conclusions can also be made regarding the assessment of circulating osteoclast precursors. These findings imply that other factors within the local joint environment, perhaps alterations in osteoblast function or expression of proinflammatory cytokines such as TNF-α, may act in concert with these soluble mediators of bone remodeling to promote osteoclastogenesis, and, in turn, bone erosion.

## Conclusions

This work has shown that systemic expression of soluble mediators of bone remodeling is disordered in PsA. Factors that promote osteoclastogenesis or inhibit osteoblast function are elevated in the circulation of patients with PsA and may contribute to periarticular bone loss in this disease. Prospective studies will be of interest to determine the role of these factors in progression of bone resorption and the effects of treatment in patients with PsA.

## Abbreviations

ANOVA: analysis of variance; BMD: bone mineral density; CASPAR: Classification of Psoriatic Arthritis; DAS: disease activity score; DEXA: dual-energy x-ray absorptiometer; DKK-1: Dikkopf-1; ELISA: enzyme-linked immunosorbent assay; M-CSF: macrophage-colony stimulating factor; MNCS: multinucleated cells; MSASSS: modified Stoke Ankylosing Spondylitis Spine Score; OPG: osteoprotegerin; PASI: Psoriasis Area and Severity Index; PBMCS: peripheral blood mononuclear cells; PNSS: Psoriasis Nail Severity Score; PSA: psoriatic arthritis; RA: rheumatoid arthritis; RANK: receptor activator of nuclear factor-κB; RANKL: receptor activator of nuclear factor-κB ligand; TNF: tumor necrosis factor; TRAP: tartrate-resistant acid phosphatase; XR: x-ray.

## Competing interests

Partial support for this study was provided by an investigator-initiated grant from Novartis. No other competing interests are declared.

## Authors' contributions

ND contributed to study conception and design, data analysis and interpretation, drafting of manuscript, and final approval of the manuscript. BP, TS, KEC, and ML contributed to acquisition and analysis of data, revision of the manuscript, and final approval of the manuscript. WJT, PJ, JC, and FMM contributed to study conception and design, data interpretation, revision of the manuscript, and final approval of the manuscript.

## References

[B1] Taylor W Gladman D Helliwell P Marchesoni A Mease P Mielants H Classification criteria for psoriatic arthritis: development of new criteria from a large international study Arthritis Rheum 2006 54 2665 2673 10.1002/art.21972 16871531

[B2] Taylor WJ Porter GG Helliwell PS Operational definitions and observer reliability of the plain radiographic features of psoriatic arthritis J Rheumatol 2003 30 2645 2658 14719209

[B3] Frediani B Allegri A Falsetti P Storri L Bisogno S Baldi F Filipponi P Marcolongo R Bone mineral density in patients with psoriatic arthritis J Rheumatol 2001 28 138 143 11196516

[B4] Ly J Pinto C Doyle A Dalbeth N McQueen FM Axial bone proliferation causing cervical myelopathy in the mutilans form of psoriatic arthritis despite peripheral bone erosion Ann Rheum Dis 2009 68 443 444 10.1136/ard.2008.093617 19213749

[B5] Teitelbaum SL Osteoclasts: what do they do and how do they do it? Am J Pathol 2007 170 427 435 10.2353/ajpath.2007.060834 17255310 PMC1851862

[B6] Yasuda H Shima N Nakagawa N Yamaguchi K Kinosaki M Mochizuki S Tomoyasu A Yano K Goto M Murakami A Tsuda E Morinaga T Higashio K Udagawa N Takahashi N Suda T Osteoclast differentiation factor is a ligand for osteoprotegerin/osteoclastogenesis-inhibitory factor and is identical to TRANCE/RANKL Proc Natl Acad Sci USA 1998 95 3597 3602 10.1073/pnas.95.7.3597 9520411 PMC19881

[B7] Lacey DL Timms E Tan HL Kelley MJ Dunstan CR Burgess T Elliott R Colombero A Elliott G Scully S Hsu H Sullivan J Hawkins N Davy E Capparelli C Eli A Qian YX Kaufman S Sarosi I Shalhoub V Senaldi G Guo J Delaney J Boyle WJ Osteoprotegerin ligand is a cytokine that regulates osteoclast differentiation and activation Cell 1998 93 165 176 10.1016/S0092-8674(00)81569-X 9568710

[B8] Bafico A Liu G Yaniv A Gazit A Aaronson SA Novel mechanism of Wnt signalling inhibition mediated by Dickkopf-1 interaction with LRP6/Arrow Nat Cell Biol 2001 3 683 686 10.1038/35083081 11433302

[B9] Tian E Zhan F Walker R Rasmussen E Ma Y Barlogie B Shaughnessy JD Jr The role of the Wnt-signaling antagonist DKK1 in the development of osteolytic lesions in multiple myeloma N Engl J Med 2003 349 2483 2494 10.1056/NEJMoa030847 14695408

[B10] Morvan F Boulukos K Clement-Lacroix P Roman S Suc-Royer I Vayssiere B Ammann P Martin P Pinho S Pognonec P Mollat P Niehrs C Baron R Rawadi G Deletion of a single allele of the Dkk1 gene leads to an increase in bone formation and bone mass J Bone Miner Res 2006 21 934 945 10.1359/jbmr.060311 16753024

[B11] Diarra D Stolina M Polzer K Zwerina J Ominsky MS Dwyer D Korb A Smolen J Hoffmann M Scheinecker C van der Heide D Landewe R Lacey D Richards WG Schett G Dickkopf-1 is a master regulator of joint remodeling Nat Med 2007 13 156 163 10.1038/nm1538 17237793

[B12] Ritchlin CT Haas-Smith SA Li P Hicks DG Schwarz EM Mechanisms of TNF-alpha- and RANKL-mediated osteoclastogenesis and bone resorption in psoriatic arthritis J Clin Invest 2003 111 821 831 12639988 10.1172/JCI16069 PMC153764

[B13] Colucci S Brunetti G Cantatore FP Oranger A Mori G Quarta L Cirulli N Mancini L Corrado A Grassi FR Grano M Lymphocytes and synovial fluid fibroblasts support osteoclastogenesis through RANKL, TNFalpha, and IL-7 in an in vitro model derived from human psoriatic arthritis J Pathol 2007 212 47 55 10.1002/path.2153 17370327

[B14] Anandarajah AP Schwarz EM Totterman S Monu J Feng CY Shao T Haas-Smith SA Ritchlin CT The effect of etanercept on osteoclast precursor frequency and enhancing bone marrow oedema in patients with psoriatic arthritis Ann Rheum Dis 2008 67 296 301 10.1136/ard.2007.076091 17967829

[B15] Chiu YG Shao T Feng C Mensah KA Thullen M Schwarz EM Ritchlin CT CD16 (FcRgammaIII) as a potential marker of osteoclast precursors in psoriatic arthritis Arthritis Res Ther 2010 12 R14 10.1186/ar2915 20102624 PMC2875642

[B16] Kitaura H Zhou P Kim HJ Novack DV Ross FP Teitelbaum SL M-CSF mediates TNF-induced inflammatory osteolysis J Clin Invest 2005 115 3418 3427 10.1172/JCI26132 16294221 PMC1283943

[B17] Kong YY Feige U Sarosi I Bolon B Tafuri A Morony S Capparelli C Li J Elliott R McCabe S Wong T Campagnuolo G Moran E Bogoch ER Van G Nguyen LT Ohashi PS Lacey DL Fish E Boyle WJ Penninger JM Activated T cells regulate bone loss and joint destruction in adjuvant arthritis through osteoprotegerin ligand Nature 1999 402 304 309 10.1038/46303 10580503

[B18] Redlich K Hayer S Maier A Dunstan CR Tohidast-Akrad M Lang S Turk B Pietschmann P Woloszczuk W Haralambous S Kollias G Steiner G Smolen JS Schett G Tumor necrosis factor alpha-mediated joint destruction is inhibited by targeting osteoclasts with osteoprotegerin Arthritis Rheum 2002 46 785 792 10.1002/art.10097 11920416

[B19] Cohen SB Dore RK Lane NE Ory PA Peterfy CG Sharp JT van der Heijde D Zhou L Tsuji W Newmark R Denosumab treatment effects on structural damage, bone mineral density, and bone turnover in rheumatoid arthritis: a twelve-month, multicenter, randomized, double-blind, placebo-controlled, phase II clinical trial Arthritis Rheum 2008 58 1299 1309 10.1002/art.23417 18438830

[B20] Fredriksson T Pettersson U Severe psoriasis: oral therapy with a new retinoid Dermatologica 1978 157 238 244 10.1159/000250839 357213

[B21] Williamson L Dalbeth N Dockerty JL Gee BC Weatherall R Wordsworth BP Extended report: nail disease in psoriatic arthritis: clinically important, potentially treatable and often overlooked Rheumatology (Oxford) 2004 43 790 794 10.1093/rheumatology/keh198 15113998

[B22] van der Heijde D Kavanaugh A Gladman DD Antoni C Krueger GG Guzzo C Zhou B Dooley LT de Vlam K Geusens P Birbara C Halter D Beutler A Infliximab inhibits progression of radiographic damage in patients with active psoriatic arthritis through one year of treatment: results from the induction and maintenance psoriatic arthritis clinical trial 2 Arthritis Rheum 2007 56 2698 2707 10.1002/art.22805 17665424

[B23] Dalbeth N Smith T Nicolson B Clark B Callon K Naot D Haskard DO McQueen FM Reid IR Cornish J Enhanced osteoclastogenesis in patients with tophaceous gout: urate crystals promote osteoclast development through interactions with stromal cells Arthritis Rheum 2008 58 1854 1865 10.1002/art.23488 18512794

[B24] Massey HM Flanagan AM Human osteoclasts derive from CD14-positive monocytes Br J Haematol 1999 106 167 170 10.1046/j.1365-2141.1999.01491.x 10444181

[B25] Shalhoub V Elliott G Chiu L Manoukian R Kelley M Hawkins N Davy E Shimamoto G Beck J Kaufman SA Van G Scully S Qi M Grisanti M Dunstan C Boyle WJ Lacey DL Characterization of osteoclast precursors in human blood Br J Haematol 2000 111 501 512 10.1046/j.1365-2141.2000.02379.x 11122091

[B26] Williamson L Dockerty JL Dalbeth N McNally E Ostlere S Wordsworth BP Clinical assessment of sacroiliitis and HLA-B27 are poor predictors of sacroiliitis diagnosed by magnetic resonance imaging in psoriatic arthritis Rheumatology (Oxford) 2004 43 85 88 10.1093/rheumatology/keg475 13130147

[B27] Docherty P Mitchell MJ MacMillan L Mosher D Barnes DC Hanly JG Magnetic resonance imaging in the detection of sacroiliitis J Rheumatol 1992 19 393 401 1578453

[B28] Daoussis D Liossis SN Solomou EE Tsanaktsi A Bounia K Karampetsou M Yiannopoulos G Andonopoulos AP Evidence that Dkk-1 is dysfunctional in ankylosing spondylitis Arthritis Rheum 2010 62 150 158 10.1002/art.27231 20039407

[B29] Yoshida H Hayashi S Kunisada T Ogawa M Nishikawa S Okamura H Sudo T Shultz LD The murine mutation osteopetrosis is in the coding region of the macrophage colony stimulating factor gene Nature 1990 345 442 444 10.1038/345442a0 2188141

[B30] Faccio R Takeshita S Zallone A Ross FP Teitelbaum SL c-Fms and the alphavbeta3 integrin collaborate during osteoclast differentiation J Clin Invest 2003 111 749 758 12618529 10.1172/JCI16924 PMC151897

[B31] Maksymowych WP Landewe R Tak PP Ritchlin CJ Ostergaard M Mease PJ El-Gabalawy H Garnero P Gladman DD Fitzgerald O Aletaha D Bykerk VP Bathon JM Syversen SW Boers M Geusens P Inman RD Kraus VB Kvien TK Taylor WJ Wells GA van der Heijde D Reappraisal of OMERACT 8 draft validation criteria for a soluble biomarker reflecting structural damage endpoints in rheumatoid arthritis, psoriatic arthritis, and spondyloarthritis: the OMERACT 9 v2 criteria J Rheumatol 2009 36 1785 1791 10.3899/jrheum.090346 19671813

[B32] Maksymowych WP Fitzgerald O Wells GA Gladman DD Landewe R Ostergaard M Taylor WJ Christensen R Tak PP Boers M Syversen SW Bathon JM Ritchlin CJ Mease PJ Bykerk VP Garnero P Geusens P El-Gabalawy H Aletaha D Inman RD Kraus VB Kvien TK van der Heijde D Proposal for levels of evidence schema for validation of a soluble biomarker reflecting damage endpoints in rheumatoid arthritis, psoriatic arthritis, and ankylosing spondylitis, and recommendations for study design J Rheumatol 2009 36 1792 1799 10.3899/jrheum090347 19671814

